# The Impact of AI Negative Feedback vs. Leader Negative Feedback on Employee Withdrawal Behavior: A Dual-Path Study of Emotion and Cognition

**DOI:** 10.3390/bs15020152

**Published:** 2025-01-30

**Authors:** Xinyue Li, Mingpeng Huang, Jialin Liu, Yifan Fan, Min Cui

**Affiliations:** School of Business, University of International Business and Economics, Beijing 100029, China; 202100322015@uibe.edu.cn (X.L.); hmp@uibe.edu.cn (M.H.); 202310320908@uibe.edu.cn (Y.F.); 202100320079@uibe.edu.cn (M.C.)

**Keywords:** AI negative feedback, leader negative feedback, shame, self-efficacy, withdrawal behavior, AI knowledge

## Abstract

In the workplace, the application of artificial intelligence (AI) is becoming increasingly widespread, including in employee performance management where AI feedback is gaining importance. Some companies are also using AI to provide negative feedback to employees. Our research compares the impact of AI negative feedback and leader negative feedback on employees. In order to explore the impact of AI negative feedback on employees, we investigated how AI negative feedback impacts employee psychology and behavior and compared these effects to those of human leader negative feedback, within the framework of the feedback process model. To explore these differences, we conducted three experimental studies (*n* = 772) from two different regions (i.e., China and the United States). The results reveal that leader negative feedback induces greater feelings of shame in employees, leading to work withdrawal behaviors, compared to AI negative feedback. Conversely, AI negative feedback has a more detrimental effect on employees’ self-efficacy, leading to work withdrawal behaviors, compared to leader negative feedback. Furthermore, employees’ AI knowledge moderates the relationship between negative feedback sources and employee withdrawal behavior. Specifically, employees who perceive themselves as having limited AI knowledge are more likely to feel ashamed when receiving leader negative feedback than when receiving AI negative feedback. Conversely, employees who believe they are knowledgeable about AI are more likely to have their self-efficacy undermined by AI negative feedback than leader negative feedback. Our research contributes significantly to the literature on AI versus human feedback and the role of feedback sources, providing practical insights for organizations on optimizing AI usage in delivering negative feedback.

## 1. Introduction

Artificial intelligence (AI) refers to machines that can perform cognitive functions typically associated with human thinking, such as learning, interacting, and problem-solving (Nilsson, 1971). The functions and roles that AI plays in the workplace are diverse, including providing feedback to employees. AI feedback refers to using AI technology to monitor, analyze, and evaluate employees’ performance in real time, offering managers insights into employee performance (Tong et al., 2021). An increasing number of companies are adopting AI to provide feedback to their employees. For example, Enaible, an AI monitoring software, gathers detailed data on employees’ work performance and generates efficiency scores based on these data (Heaven, 2020). Research indicates that feedback significantly affects employees’ psychological states and behaviors (Aleassa, 2014). However, research on AI feedback remains in its early stages, and the underlying mechanisms through which AI feedback influences psychological and behavioral outcomes remain underexplored (Tong et al., 2021).

Current research on AI feedback (vs. human feedback) primarily focuses on its positive impact on employees. For instance, AI feedback, being more personalized than human feedback, significantly enhances employee performance (Hoffmann & Thommes, 2020). It is perceived as more accurate and fair, fostering commitment and satisfaction while reducing turnover (Qin et al., 2023; Riaz & Ghanghas, 2024). Additionally, AI negative feedback promotes promotion-focused cognition over prevention-focused cognition, further improving job performance (Pei et al., 2024). However, the potential negative impacts of AI feedback remain underexplored. Negative feedback is inherently more complex in its effects on employees compared to positive feedback (Ilgen & Davis, 2000; Kluger & DeNisi, 1996), necessitating detailed research on the processes and outcomes of employee responses to such feedback. This raises critical questions: Is negative feedback from AI always more effective than that from human leaders? How does AI-provided negative feedback influence employee psychology and behavior, and how do these effects differ from human leader feedback? Addressing these questions can deepen the understanding of employees’ cognitive, emotional, and behavioral responses to negative feedback during human–AI interactions. This, in turn, enriches the theoretical framework of AI feedback and aids organizations in leveraging AI feedback more effectively. Previous research indicates that employees often respond to negative feedback with withdrawal behaviors (Belschak & Den Hartog, 2009; Xing et al., 2021). Accordingly, this research focuses on comparing the effects of negative feedback from AI and human leaders on employees’ withdrawal behaviors, incorporating both emotional and cognitive dimensions.

We draw on the feedback process model (Ilgen et al., 1979) to develop a theoretical model comparing the effects of two negative feedback sources (AI vs. human leader) on employee withdrawal behavior through employee feelings of shame and self-efficacy. The feedback process model suggests that individuals, upon receiving feedback, first engage in emotional and cognitive processing, which then informs their response behaviors (Ilgen et al., 1979). Moreover, the feedback source can influence how individuals process the feedback (Ilgen et al., 1979). We propose that leader negative feedback induces stronger feelings of shame in employees compared to AI negative feedback, which can lead to withdrawal behaviors (Bono & Ilies, 2006; Mawritz et al., 2014). Additionally, AI negative feedback reduces employees’ self-efficacy to a greater extent than leader negative feedback, which can lower employees’ self-efficacy and lead to withdrawal behaviors.

Furthermore, the feedback process model also posits that the recipient’s perception of the feedback provider influences their psychological and behavioral responses to the feedback (Leung et al., 2001; Nae et al., 2015). AI knowledge, which refers to an individual’s understanding of AI, reflects their perception of AI (Chiu et al., 2021). We argue that AI knowledge may influence their responses to different negative feedback providers. Specifically, we propose that employees who perceive themselves as less knowledgeable about AI are more likely to experience shame when receiving negative feedback from a leader compared to receiving negative feedback from AI. On the other hand, employees who consider themselves more knowledgeable about AI are more likely to have their self-efficacy undermined when receiving negative feedback from AI compared to receiving negative feedback from a leader.

Overall, our research has made significant contributions to the literature on AI and human feedback sources. First, we have revealed the negative effects of AI negative feedback on employees by comparing its differential impact with leader negative feedback. Second, we have advanced the literature on feedback sources by addressing the question of the differential impact of negative feedback provided by leaders and AI on employees. The emergence of AI has disrupted people’s perceptions of feedback sources (Lechermeier & Fassnacht, 2018; Tong et al., 2021). This research responds to scholars’ calls to clarify the impact and mechanisms of AI as a feedback source on employees (Lechermeier & Fassnacht, 2018) and highlights the differences among various feedback sources (Herold et al., 1987). Third, we highlight the heterogeneity in employees’ perceptions of negative feedback sources, reveal the moderating effect of employees’ AI knowledge, and specify the boundary conditions of the impact of negative feedback sources (AI vs. leaders) on employees. These contributions have practical implications for organizations to effectively utilize AI feedback functions.

## 2. Theory and Hypotheses

### 2.1. Impact of Negative Feedback Source on Employees’ Feeling of Shame and Employees’ Self-Efficacy

Ilgen et al. (1979) proposed the feedback process model, which posits that individuals first process feedback through emotional and cognitive reactions before responding to it. Ilgen and colleagues also break down the feedback process into three components: the feedback source, feedback information, and feedback receiver, each of which can influence an employee’ s psychological response to feedback. Recently, companies have begun using AI to assist in employee performance evaluations and feedback processes (Heaven, 2020; Roose, 2019; Marr, 2018), meaning employees may now receive feedback from both their leaders and AI. Since leaders and AI are distinct feedback sources, they may have differing effects on employees’ emotions, cognition, and behaviors (Ilgen et al., 1979).

Negative feedback refers to the guidance, criticism, or corrective actions given to employees when their performance or behavior falls short of expectations or standards (Ilies & Judge, 2005). With the growing integration of AI technology, many companies are increasingly using AI to deliver such feedback, including negative evaluations. This raises the question of whether negative feedback from AI has a different impact on employees compared to negative feedback from human leaders. According to the feedback process model, we posit that negative feedback from leaders is more likely to evoke feelings of shame in employees than feedback from AI. Shame is a painful self-conscious emotion that stems from self-perceived failures to meet external expectations, such as receiving criticism from a leader, experiencing task failure, or making mistakes (Daniels & Robinson, 2019). When employees receive negative feedback, they often engage in negative self-evaluation, perceiving themselves as inadequate or underperforming, which can lead to shame (Belschak & Den Hartog, 2009; Xing et al., 2021). Compared to AI negative feedback, leader negative feedback involves more complex interpersonal dynamics, making employees more emotionally vulnerable to such feedback (Mawritz et al., 2014). Leader negative feedback is typically more emotionally charged and personalized, heightening the likelihood of triggering shame (Bono & Ilies, 2006). In contrast, AI negative feedback is often perceived as more neutral and objective, given its data-driven, algorithm-based nature (Zappone et al., 2019). Because AI lacks personal involvement or emotional nuance (Liu-Thompkins et al., 2022), employees may interpret AI feedback as an impartial evaluation, reducing the likelihood of experiencing shame. Consequently, employees are more likely to perceive AI negative feedback as a neutral and objective evaluation, rather than feeling shame, as they might with feedback from leaders. Therefore, we hypothesize

**H1.**  *Compared to negative feedback from AI, negative feedback from leaders is more effective in increasing employees’ feeling of shame.*

Furthermore, we posit that negative feedback from AI is more likely to diminish employees’ self-efficacy compared to negative feedback from human leaders. Self-efficacy refers to an individual’s belief in their ability to successfully perform tasks, reflecting their self-assessment and level of confidence in their abilities (Bandura, 1977). Negative feedback can trigger self-doubt, thereby lowering self-efficacy (Parker et al., 2016; Kluger & DeNisi, 1996). First, AI feedback is often perceived as highly data-driven and fact-based, leading employees to view it as more accurate and objective (Shin & Park, 2019; Sundar & Nass, 2001). This perceived accuracy can intensify self-doubt. In contrast, leader feedback may not be as comprehensive or objective, as it may fail to cover all aspects of performance. As a result, negative feedback from leaders may not reduce employees’ self-efficacy to the same extent as AI negative feedback. Second, AI feedback is typically viewed as free from personal biases, such as emotions or subjective preferences, which can influence human feedback (Gray & Wegner, 2012). AI follows a consistent and algorithmic process; it is perceived as impartial and devoid of personal factors (Raisch & Krakowski, 2021). This perceived objectivity of AI negative feedback may further undermine employees’ confidence in their abilities, leading to a greater reduction in self-efficacy than leader negative feedback. Therefore, we hypothesize

**H2.**  *Compared to negative feedback from leaders, negative feedback from AI is more effective in reducing employees’ self-efficacy.*

### 2.2. Negative Feedback Source, Employees’ Feeling of Shame, Employees’ Self-Efficacy, and Employee Withdrawal Behavior

The feedback process model posits that emotional responses following feedback can stimulate corresponding behaviors (Ilgen et al., 1979). Work withdrawal behaviors refer to proactive actions employees take to avoid or express dissatisfaction with their work environment, ranging from occasional daydreaming to more severe behaviors like tardiness, absenteeism, and, ultimately, resignation (Berry et al., 2012; Gupta & Jenkins, 1991). We suggest that employees’ feelings of shame can significantly influence their work withdrawal behaviors. Shame is an action-oriented emotional response triggered by specific stimuli (O’Reilly et al., 2016), typically arising when individuals perceive themselves as failing to meet others’ expectations. This may occur in situations such as task failures, workplace mistakes, or criticism from superiors, resulting in a painful self-awareness (Daniels & Robinson, 2019). To protect themselves from further emotional distress, employees experiencing shame may adopt avoidance strategies (Daniels & Robinson, 2019; de Hooge et al., 2010), which can manifest in work withdrawal behaviors, such as avoiding contact, unexcused absenteeism, or silence (Peng et al., 2019). Therefore, we propose that employees’ feelings of shame may lead to increased withdrawal behaviors.

Based on the above discussion, we propose that employees’ feeling of shame positively affects work withdrawal behavior. In conjunction with Hypothesis 1, we further believe that negative feedback from leaders, as opposed to negative feedback from AI, has a more significant indirect impact on employees’ work withdrawal behavior by increasing their feeling of shame. Therefore, we propose the following hypothesis:

**H3.**  *The indirect effect of negative feedback source on employees’ withdrawal behavior occurs via employees’ feeling of shame. Specifically, compared to negative feedback from AI, negative feedback from leaders is more effective in increasing employees’ withdrawal behavior via employees’ feeling of shame.*

In addition to emotional experiences, employees’ self-efficacy also plays a crucial role in shaping their work withdrawal behaviors. Self-efficacy, which influences individuals’ behavioral choices, directs their actions and responses to challenges (Heuven et al., 2006). First, employees with low self-efficacy may feel powerless to alter their circumstances, becoming more focused on the potential consequences of failure (Bandura, 1983). When they believe their efforts cannot restore their self-worth, they are more likely to adopt withdrawal or avoidance strategies to mitigate further perceived failure or harm (Daniels & Robinson, 2019; de Hooge et al., 2010). Second, low self-efficacy often fosters pessimism about achieving success, resulting in diminished motivation to overcome difficulties or pursue challenges (Abele & Spurk, 2009; Bandalos et al., 1995). Employees who lack confidence in their ability to succeed may avoid development opportunities, contributing to work withdrawal behaviors (Pingel et al., 2019).

Based on the above discussion, we propose that employees’ self-efficacy negatively affects work withdrawal behavior. In conjunction with Hypothesis 2, we further propose that negative feedback from AI, as opposed to negative feedback from a leader, has a more significant indirect impact on employees’ work withdrawal behavior by reducing their self-efficacy. Therefore, we propose the following hypothesis:

**H4**  . *The indirect effect of negative feedback source on employees’ withdrawal behavior occurs via employees’ self-efficacy. Specifically, compared to negative feedback from leaders, negative feedback from AI is more effective in increasing employees’ withdrawal behavior via employees’ self-efficacy.*

### 2.3. Moderating Effect of Employee AI Knowledge

The feedback process model suggests that the recipient’s perception of the feedback provider influences their psychological and behavioral responses to feedback (Leung et al., 2001; Nae et al., 2015). Employees’ AI knowledge—defined as their perceived understanding of AI (Chiu et al., 2021)—may moderate their responses to negative feedback from different sources. We propose that employees’ AI knowledge moderates the relationship between negative feedback sources and feelings of shame. Specifically, negative feedback from leaders is more likely to elicit feelings of shame when employees perceive their AI knowledge to be low, compared to negative feedback from AI. However, when employees perceive themselves to have high AI knowledge, the source of negative feedback may have a diminished impact on their feelings of shame.

Research shows that increased knowledge can alter beliefs and influence attitudes and behaviors toward new technologies (He et al., 2024). We posit that a greater subjective understanding of AI leads employees to place greater value on AI, thereby intensifying the feelings of shame induced by AI negative feedback. Employees with high AI knowledge are likely to have more emotionally charged attitudes—whether positive or negative—toward AI (He et al., 2024), which leads them to value AI feedback more highly. As a result, negative feedback from AI triggers a negative emotional response comparable to that of leader negative feedback. Conversely, employees with low AI knowledge may struggle to distinguish between fiction and reality and tend to hold less pronounced emotional attitudes toward AI (He et al., 2024). This makes them less sensitive to AI negative feedback, meaning they are less likely to experience the same level of shame from AI negative feedback as they would from leader negative feedback. Therefore, we hypothesize

**H5.**  *AI knowledge moderates the relationship between negative feedback source and employees’ feeling of shame. Specifically, when employees have low AI knowledge, compared to negative feedback from AI, negative feedback from leaders is more effective in increasing employees’ feeling of shame. However, when employees have high AI knowledge, the impact of negative feedback source on employees’ feeling of shame is not significant.*

The credibility of the feedback provider is an important factor in influencing feedback response (Giffin, 1967; Nae et al., 2015). Feedback from a provider perceived as reliable and trustworthy is more likely to be regarded as useful and effective (Nae et al., 2015). We propose that employees’ AI knowledge moderates the impact of negative feedback sources on their self-efficacy. Specifically, negative feedback from AI is more likely to reduce self-efficacy when employees perceive themselves as having high AI knowledge, compared to negative feedback from leaders. However, when employees have low AI knowledge, the source of negative feedback—whether from AI or leaders—does not significantly impact their self-efficacy.

Research on AI knowledge suggests that understanding AI includes awareness of its current development capabilities (Chiu et al., 2021). AI systems, which are trained on vast amounts of data and powered by algorithms, are often viewed as processing information without bias (Raisch & Krakowski, 2021). Employees with higher AI knowledge are more likely to recognize these objective and accurate attributes, leading them to view AI feedback as more reliable and trustworthy (Nae et al., 2015)^1^. Therefore, when receiving negative feedback from AI, employees with high AI knowledge are more likely to have their confidence undermined, leading to reduced self-efficacy. In contrast, employees with low AI knowledge, who may lack an understanding of AI’s development and capabilities, are less likely to fully recognize its objective and accurate attributes. As a result, these employees are less inclined to perceive AI feedback as reliable or trustworthy. Consequently, the source of negative feedback—whether AI or leaders—does not have a significant differential effect on their self-efficacy. Therefore, we hypothesize

**H6.**  *Employees’ AI knowledge moderates the relationship between negative feedback source and employees’ self-efficacy. Specifically, when employees have high AI knowledge, compared to negative feedback from leaders, negative feedback from AI is more effective in reducing employees’ self-efficacy. However, when employees have low AI knowledge, the impact of negative feedback source on employees’ self-efficacy is not significant.*

Integrating our proposed moderating effect of employee AI knowledge between negative feedback sources and employees’ feeling of shame (Hypothesis 5) with the indirect effect of negative feedback sources on employees’ withdrawal behavior through employees’ feeling of shame (Hypothesis 3), we hypothesize

**H7.**  *Employees’ AI knowledge moderates the indirect effect of negative feedback source on employees’ withdrawal behavior via employees’ feeling of shame such that the indirect effect is stronger when employees’ AI knowledge is low (vs. high).*

Integrating our proposed moderating effect of employee AI knowledge between negative feedback sources and employees’ self-efficacy (Hypothesis 6) with the indirect effect of negative feedback sources on employees’ withdrawal behavior through employees’ self-efficacy (Hypothesis 4), we hypothesize

**H8.**  *Employees’ AI knowledge moderates the indirect effect of negative feedback source on employees’ withdrawal behavior via employees’ self-efficacy such that the indirect effect is stronger when employees’ AI knowledge is high (vs. low).*

**Figure 1:**
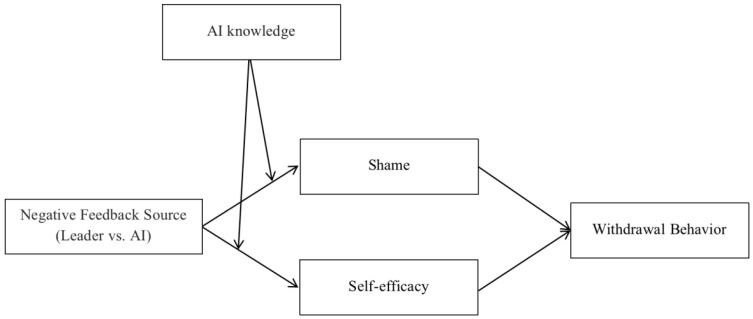
Proposed theoretical model.

In summary, the research model is visually represented in Figure 1.

## 3. Overview of Studies

We tested our hypotheses in three studies. Study 1 was an experimental study with full-time employees from various industries in China as participants. Study 2 used similar experimental materials as Study 1, but the participants were full-time employees from the United States. Study 3 was also an experimental study with participants from China. In contrast to Study 2, it employed a 2 × 2 between-group design and incorporated a more immersive experimental scenario. Unlike the experimental scenarios in Study 1 and Study 2, we enhanced the external validity of the study by having participants complete a real task and providing them with negative feedback from different sources based on this task. Data were analyzed using G*Power v.3.1 and SPSS v.26. The pre-registration material is available at https://aspredicted.org/3MM_V3B, accessed on 12 October 2023.

## 4. Study 1 Method

### 4.1. Participants and Procedures

In Study 1, we recruited participants from China through the questionnaire collection platform Credamo (https://www.credamo.com/#/, accessed on 1 January 2024) (Jiang et al., 2022; Ma et al., 2023). We invited working Chinese employees who met the following criteria: they were over 18 years old, employed full-time, and had a questionnaire completion rate of at least 90% on Credamo. Before data collection, we conducted a priori power analysis with G*Power v.3.1 (Faul et al., 2007). The result showed that to reach 0.80 power, based on a medium effect size f = 0.25 and a significant level of 0.05, 128 participants were needed. We initially recruited 304 participants to account for the attrition of careless or incomplete responses. Each participant was compensated with RMB 3 (approximately USD 0.42). In order to screen careless responses, we took the suggestion of Meade and Craig (2012) and added a basic attention check item (e.g., “Please choose ‘Strongly disagree’ for this question”). We excluded 11 participants who failed the attention check. The final sample included 293 participants. Among the 293 participants, 175 were female (59.73%), with an average age of 31.92 years (SD = 9.65). They also had an average education of 16.63 years (SD = 2.35), and their average organizational tenure was 7.61 years (SD = 7.10).

Participants were randomly assigned to one of two conditions: negative feedback from the immediate supervisor condition and negative feedback from AI condition. The participants were instructed to read a vignette in which they were required to play the role of an employee at Beta Media Company. Referring to the experimental design of Hoever et al. (2018), we used a task performance ranking in the bottom 30% of the department as a description of negative feedback.

### 4.2. Manipulations

Negative feedback source. In the leader negative feedback condition (*n* = 143), participants read the following:

*You are an employee at Beta Media Company, and your immediate supervisor evaluates your job performance, including work progress, task completion, and work quality. Your immediate supervisor generates performance evaluation reports for each employee based on their monthly performance. At the end of the month, you receive a performance evaluation report from your immediate supervisor, which states the following:*

*“This month, you failed to deliver work results on time, and the quality of the delivered work was poor, with significant errors and defects. Taking into account your overall performance in terms of work progress, task completion, and work quality, your work performance this month ranks in the bottom 30% of the department.”*

In the AI negative feedback condition (*n* = 150), participants read the following:

*You are an employee at Beta Media Company, which has introduced an Artificial Intelligence (AI) system to assess employees’ job performance, including work progress, task completion, and work quality. The AI system generates performance evaluation reports for each employee based on their monthly performance. At the end of the month, you receive a performance evaluation report from the AI system, which states the following:*

*“This month, you failed to deliver work results on time, and the quality of the delivered work was poor, with significant errors and defects. Taking into account your overall performance in terms of work progress, task completion, and work quality, your work performance this month ranks in the bottom 30% of the department.”*

### 4.3. Measures

Employees’ feeling of shame was assessed using the three-item scale from Fredrickson et al. (2003) on a 5-point scale (1 = not at all; 5 = extremely). We asked the participants to indicate “how do you feel after receiving the evaluation report?” The items were “ashamed”, “humiliated”, and “disgraced”. The Cronbach’s α of this study was 0.82.

Employees’ self-efficacy was assessed using the three-item scale from Spreitzer (1995) on a 5-point scale (1 = strongly disagree; 5 = strongly agree). We asked the participants to indicate “to what extent do you agree or disagree with the following statements after receiving the evaluation report?” A sample item is “I am confident about my ability to do my jobs”. The Cronbach’s α of this study was 0.75.

Negative feedback source manipulation check. After participants completed all the questions, we asked them to indicate whether their performance evaluation report was provided by their immediate supervisor or an AI system (0 = the immediate supervisor; 1 = AI system).

## 5. Study 1 Results

### 5.1. Manipulation Check

The negative feedback source manipulation check was higher in the negative feedback from AI condition (M = 0.90, SD = 0.30) than in the negative feedback from leaders condition (M = 0.10, SD = 0.31, F _[1, 291]_ = 500.17, *p* < 0.001, η^2^ = 0.63). Our manipulation was effective.

### 5.2. Tests of Hypotheses

The analysis of variance (ANOVA) results revealed that participants assigned to the negative feedback from leaders condition rated significantly higher levels of employees’ feeling of shame (M = 3.59, SD = 1.03) compared with those assigned to the negative feedback from AI condition (M = 3.34, SD = 1.10; F _[1, 291]_ = 4.20, *p* < 0.05, η^2^ = 0.01). Thus, Hypothesis 1 was supported. Furthermore, participants assigned to the negative feedback from AI condition rated significantly lower levels of employees’ self-efficacy (M = 2.37, SD = 0.90) compared with those assigned to the negative feedback from leaders condition (M = 2.61, SD = 1.11; F _[1, 291]_ = 4.13, *p* < 0.05, η^2^ = 0.03). Thus, Hypothesis 2 was supported.

In sum, Study 1 validated the relationship between negative feedback source and employees’ feeling of shame, and the relationship between negative feedback source and employees’ self-efficacy. However, Study 1 only tested part of our theoretical model and recruited only Chinese participants. To address these limitations, we conducted Study 2 to test all the hypotheses.

## 6. Study 2 Method

### 6.1. Participants and Procedures

In Study 2, we recruited participants in the United States from Prolific Academic (https://www.prolific.co/, accessed on 1 January 2024) (Palan & Schitter, 2018; Park et al., 2022). Eligible participants were required to be over 18 years old, employed full-time, and have a questionnaire completion rate of at least 95% on Prolific. Before data collection, we conducted a priori power analysis with G*Power v.3.1 (Faul et al., 2007). The result showed that to reach 0.80 power, based on a medium effect size f = 0.25 and a significant level of 0.05, 128 participants were needed. We initially recruited 219 participants to account for the attrition of careless or incomplete responses. Each participant was compensated with USD 0.65. We used the same method as in Study 1 to screen careless responses. We excluded 12 participants who failed the attention check. The final sample included 207 participants. Among the 207 participants, 94 were female (45.41%), 63.29% were Caucasian, 14.01% were African American, 10.63% were Asian, 8.70% were Hispanic American, and 3.38% did not indicate their races. They had an average age of 38.70 years (SD = 10.20). They also had an average education of 15.70 years (SD = 3.02), and their average organizational tenure was 6.99 years (SD = 6.53).

As in Study 1, participants were randomly assigned to one of two conditions: negative feedback from the immediate supervisor condition and negative feedback from AI condition. However, in this study, we measured individuals’ AI knowledge and assessed participants’ withdrawal behavior after receiving negative feedback.

### 6.2. Manipulations

Negative feedback source. The manipulation procedure for negative feedback sources remained consistent with Study 1. In the leader negative feedback condition, there were 106 participants. In the AI negative feedback condition, there were 101 participants.

### 6.3. Measures

Employees’ AI knowledge. We measured employee’s AI knowledge using a five-item scale developed by Chiu et al. (2021) (1 = strongly disagree; 5 = strongly agree). A sample item is “I know pretty much about AI”. The Cronbach’s α of this study was 0.90.

Employees’ feeling of shame. We used the same three-item scale used in Study 1. The Cronbach’s α of this study was 0.87.

Employees’ self-efficacy. We used the same three-item scale used in Study 1. The Cronbach’s α of this study was 0.93.

Employees’ withdrawal behavior was assessed using the four-item from Lehman and Simpson (1992) on a 5-point scale (1 = very unlikely; 5 = very likely). A sample item is “I will have thoughts of being absent”. The Cronbach’s α of this study was 0.81.

Negative feedback source manipulation check. We used the same manipulation check as Study 1.

Negative feedback manipulation check. To ensure that the manipulation of negative feedback had worked as intended, we asked them to report how they felt about the feedback they received on a scale from 1 (extremely negative) to 5 (extremely positive).

## 7. Study 2 Results

### 7.1. Manipulations Check

The negative feedback source manipulation check was higher in the negative feedback from AI condition (M = 0.88, SD = 0.33) than in the negative feedback from leaders condition (M = 0.07, SD = 0.25, F _[1, 205]_ = 411.71, *p* < 0.001, η^2^ = 0.67). In addition, participants reported that feedback they received was negative (M = 1.89, SD = 1.04). Thus, our manipulations were effective.

### 7.2. Tests of Hypotheses

The analysis of variance (ANOVA) results revealed that participants assigned to the negative feedback from leaders condition rated significantly higher levels of employees’ feeling of shame (M = 3.47, SD = 0.91) compared with those assigned to the negative feedback from AI condition (M = 3.05, SD = 0.98; F _[1, 205]_ = 10.19, *p* < 0.01, η^2^ = 0.05). Thus, Hypothesis 1 was supported. Furthermore, participants assigned to the negative feedback from AI condition rated significantly lower levels of employees’ self-efficacy (M = 2.63, SD = 0.98) compared with those assigned to the negative feedback from leaders condition (M = 3.45, SD = 1.09; F _[1, 205]_ = 32.85, *p* < 0.001, η^2^ = 0.14). Thus, Hypothesis 2 was supported.

We used SPSS 26.0 Hayes PROCESS (Model 4) to verify the indirect effect. After 5000 Bootstrap samples, we found that the indirect effect of negative feedback source on employees’ withdrawal behavior via employees’ feeling of shame was significant. The indirect effect of employees’ feeling of shame was −0.10 (95% Boot CI = [−0.18, −0.03]), thus supporting Hypothesis 3. We also found that the indirect effect of negative feedback source on employees’ withdrawal behavior via employees’ self-efficacy was significant. The indirect effect of employees’ feeling of shame was 0.12 (95% Boot CI = [0.03, 0.25]), thus supporting Hypothesis 4.

**Figure 2:**
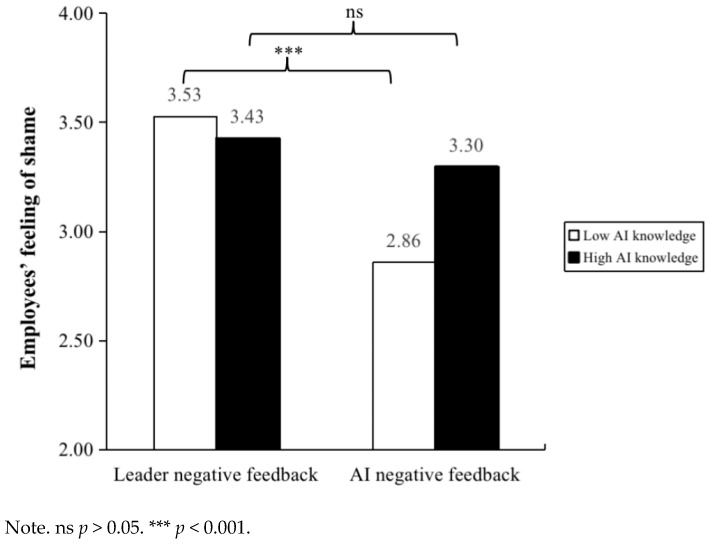
The moderating effect of employee AI knowledge between negative feedback source and employees’ feeling of shame in Study 2.

**Figure 3:**
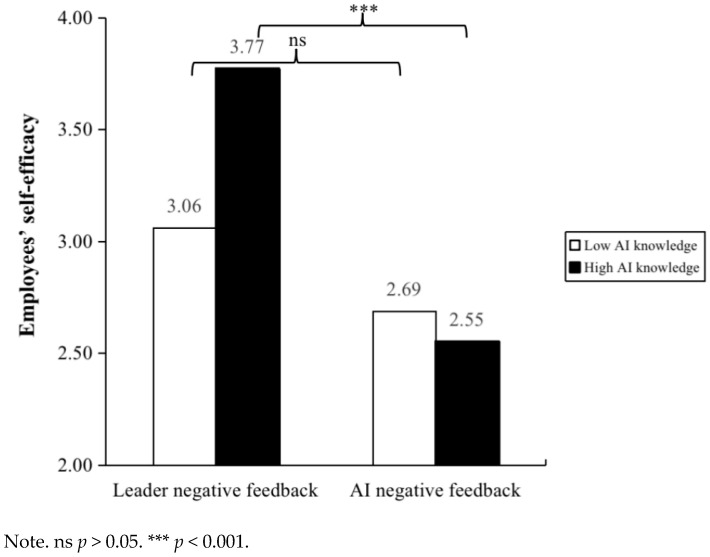
The moderating effect of employee AI knowledge between negative feedback source and employees’ self-efficacy in Study 2.

Then, we used SPSS 26.0 Hayes PROCESS (Model 1) to verify the moderation effect. As shown in Model 2 of Table 1, employees’ AI knowledge significantly moderated the relationship between negative feedback source and employees’ feeling of shame (*β* = 0.29, *p* < 0.05). We plot this moderation effect in Figure 2. The results of the conditional effects analysis showed that when employees have low AI knowledge, compared to negative feedback from AI, negative feedback from leaders is more effective in increasing employees’ feeling of shame (*β* = −0.67, 95% Boot CI = [−1.04, −0.30]). Meanwhile, when employees have high AI knowledge, the impact of negative feedback source on employees’ feeling of shame is not significant (*β* = −0.13, 95% Boot CI = [−0.50, 0.23]). Therefore, Hypothesis 5 was supported. As shown in Model 4 of Table 1, employees’ AI knowledge significantly moderated the relationship between negative feedback source and employees’ self-efficacy (*β* = −0.45, *p* < 0.01). We plot this moderation effect in Figure 3. The results of the conditional effects analysis showed that when employees have high AI knowledge, compared to negative feedback from leaders, negative feedback from AI is more effective in reducing employees’ self-efficacy (*β* = −1.22, 95% Boot CI = [−1.61, −0.82]). Meanwhile, when employees have low AI knowledge, the impact of negative feedback source on employees’ self-efficacy is not significant (*β* = −0.37, 95% Boot CI = [−0.77, 0.02]). Therefore, Hypothesis 6 was supported.

We used SPSS 26.0 Hayes PROCESS (Model 7) to assess the conditional indirect effect of AI knowledge on the relationship between negative feedback source and employees’ withdrawal behavior via employees’ feeling of shame at low (−1 SD) and high (+1 SD) values of AI knowledge. The indirect effect was −0.16 (95% Boot CI = [−0.28, −0.06]) when employees’ AI knowledge was low, versus −0.03 (95% Boot CI = [−0.12, 0.05]) when employees’ AI knowledge was high. The index of moderated mediation was 0.07 (95% Boot CI = [0.01, 0.15]). Therefore, Hypothesis 7 was supported. We assessed the conditional indirect effect of AI knowledge on the relationship between negative feedback source and employees’ withdrawal behavior via employees’ self-efficacy at low (−1 SD) and high (+1 SD) values of AI knowledge. The indirect effect was 0.18 (95% Boot CI = [0.03, 0.36]) when employees’ AI knowledge was high, versus 0.06 (95% Boot CI = [−0.02, 0.15]), when employees’ AI knowledge was low. The index of moderated mediation was 0.07 (95% Boot CI = [0.01, 0.16]). Therefore, Hypothesis 8 was supported.

In sum, Study 2 tested all the hypotheses. In Study 3, we designed a between-subjects experiment with a 2 (leader negative feedback condition vs. AI negative feedback condition) × 2 (low AI knowledge condition vs. high AI knowledge condition) between-subjects design. Furthermore, we designed more immersive scenarios in Study 3 to enhance external validity.

## 8. Study 3 Method

### 8.1. Participants and Procedures

In Study 3, we recruited participants from China through the questionnaire collection platform Credamo (https://www.credamo.com/#/, accessed on 1 January 2024). We invited working Chinese employees who met the following criteria: they were over 18 years old, employed full-time, and had a questionnaire completion rate of at least 90% on Credamo. Before data collection, we conducted a priori power analysis with G*Power v.3.1 (Faul et al., 2007). The result showed that to reach 0.80 power, based on a medium effect size f = 0.25 and a significant level of 0.05, 128 participants were needed. We initially recruited 292 participants to account for the attrition of careless or incomplete responses. Each participant was compensated with RMB 3 (approximately USD 0.42). We used the same method as in Study 1 and Study 2 to screen careless responses. We excluded 20 participants who failed the attention check. The final sample included 272 participants. Among the 272 participants, 158 were female (58.09%), with an average age of 30.06 years (SD = 8.53). They also had an average education of 16.29 years (SD = 1.68), and their average organizational tenure was 6.80 years (SD = 6.97).

Participants were randomly assigned to one of four conditions in a 2 (leader negative feedback condition vs. AI negative feedback condition) × 2 (low AI knowledge condition vs. high AI knowledge condition) between-subjects design. We first had the participants complete a test on AI and manipulated their individual AI knowledge by providing them with false feedback. Afterwards, they were asked to complete a creative task and were given false negative feedback from the immediate leader or the AI system.

### 8.2. Manipulations

**Individual AI knowledge manipulation.** In order to manipulate individuals’ AI knowledge, we used Moorman et al.’s (2004) method of subjective knowledge manipulation. Participants were asked to complete a test assessing their actual knowledge of AI. The test questions did not involve any objective or factual statements about AI, and were presented as indefinite questions, which to some extent increased the uncertainty of answering. The test materials are available on the Open Science Framework: https://osf.io/9nkux/?view_only=c3f1b6214d454212b391c73242d89c8e (accessed on 18 April 2024). Afterwards, participants received false feedback about their performance. The low-AI-knowledge group was informed that their score was better than only 10% of the test participants (*n* = 138), while the high-AI-knowledge group was told that their score was better than 90% of the test participants (*n* = 134). Subsequently, participants were asked to rate their AI knowledge (Chiu et al., 2021).

Negative feedback source manipulation. We first had participants complete a creative task (Kim & Kim, 2020), which was as follows:

*You are an employee of Company C, and the company is currently planning to create a work coffee area. In order to provide employees with more convenience in their work, the company is requesting all staff members to contribute as many creative ideas as possible for the functional design of the “work coffee area”.*

Afterwards, we used Zingoni and Byron’s (2017) negative feedback manipulation and created two conditions: leader negative feedback and AI negative feedback. In the leader negative feedback condition (*n* = 135), participants read the following:

*Your immediate supervisor, Manager Wang, will provide feedback on your performance for this task. Manager Wang’s feedback states, “After reviewing the results you submitted, your performance ranks in the bottom 30% for this creativity task. I recommend that you enrich your knowledge base, cultivate keen observation skills, and foster a rich imagination in your future work.”*

In the AI negative feedback condition (*n* = 137), participants read the following:

*The company has introduced an Artificial Intelligence (AI) system to provide feedback on your performance for this task. The AI system’s feedback is as follows: After reviewing the results you submitted, your performance ranks in the bottom 30% for this creativity task. It is recommended that you enrich your knowledge base, cultivate keen observation skills, and foster a rich imagination in your future work.*

### 8.3. Measures

Employees’ feeling of shame. We used the same three-item scale used in Study 1 and Study 2. The Cronbach’s α of this study was 0.80.

Employees’ self-efficacy. As the participants completed a creative task, we assessed their individual creative self-efficacy. We used the four-item scale from Tierney and Farmer (2011) on a 5-point scale (1 = strongly disagree; 5 = strongly agree). A sample item is “I have confidence in my ability to solve problems”. The Cronbach’s α of this study was 0.90.

Employees’ withdrawal behavior. We used the same four-item scale used in Study 2. The Cronbach’s α of this study was 0.88.

Negative feedback source manipulation check. We used the same manipulation check as Study 1 and Study 2.

Employees’ AI knowledge manipulation check. We used the same five-item scale used in Study 2. The Cronbach’s α of this study was 0.95.

Negative feedback manipulation check. We used the same manipulation check as Study 2.

## 9. Study 3 Results

### 9.1. Manipulations

The negative feedback source manipulation check was higher in the negative feedback from AI condition (M = 1.00, SD = 0.00) than in the negative feedback from leaders condition (M = 0.01, SD = 0.09, F _[1, 270]_ = 18,223.02, *p* < 0.001, η^2^ = 0.67). The employees’ AI knowledge manipulation check was higher in the high employees’ AI knowledge condition (M = 4.14, SD = 0.61) than in the low employees’ AI knowledge condition (M = 2.18, SD = 0.92, F _[1, 270]_ = 427.46, *p* < 0.001, η^2^ = 0.61). In addition, participants reported that the feedback they received was negative (M = 2.28, SD = 1.05). Thus, our manipulations were successful.

### 9.2. Tests of Hypotheses

The analysis of variance (ANOVA) results revealed that participants assigned to the negative feedback from leaders condition rated significantly higher levels of employees’ feeling of shame (M = 3.04, SD = 1.11) compared with those assigned to the negative feedback from AI condition (M = 2.67, SD = 0.77; F _[1, 270]_ = 10.46, *p* < 0.001, η^2^ = 0.04). Thus, Hypothesis 1 was supported. The analysis of variance (ANOVA) results revealed that participants assigned to the negative feedback from AI condition rated significantly lower levels of employees’ self-efficacy (M = 2.46, SD = 0.87) compared with those assigned to the negative feedback from leaders condition (M = 2.83, SD = 1.11; F _[1, 270]_ = 9.31, *p* < 0.01, η^2^ = 0.03). Thus, Hypothesis 2 was supported.

We used SPSS 26.0 Hayes PROCESS (Model 4) to verify the indirect effect. After 5000 Bootstrap samples, we found that the indirect effect of negative feedback source on employees’ withdrawal behavior via employees’ feeling of shame was significant. The indirect effect of employees’ feeling of shame was −0.09 (95% Boot CI = [−0.18, −0.02]), thus supporting Hypothesis 3. We also found that the indirect effect of negative feedback source on employees’ withdrawal behavior via employees’ self-efficacy was significant. The indirect effect of employees’ feeling of shame was 0.08 (95% Boot CI = [0.02, 0.15]), thus supporting Hypothesis 4.

**Figure 4:**
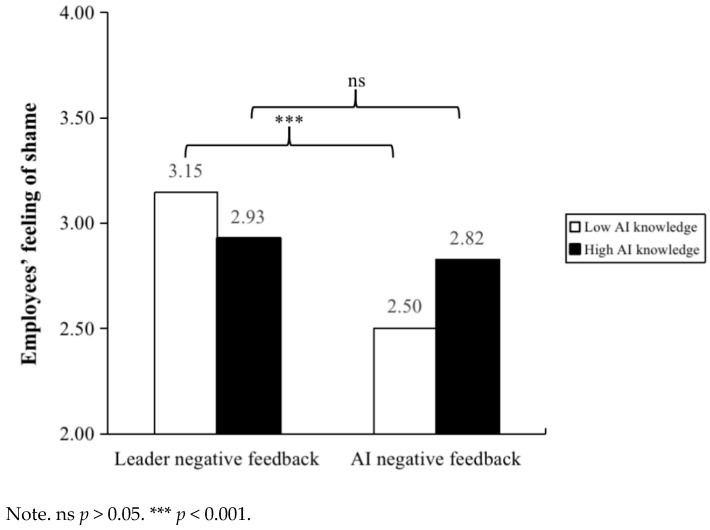
The moderating effect of employee AI knowledge between negative feedback source and employees’ feeling of shame in Study 3.

**Figure 5:**
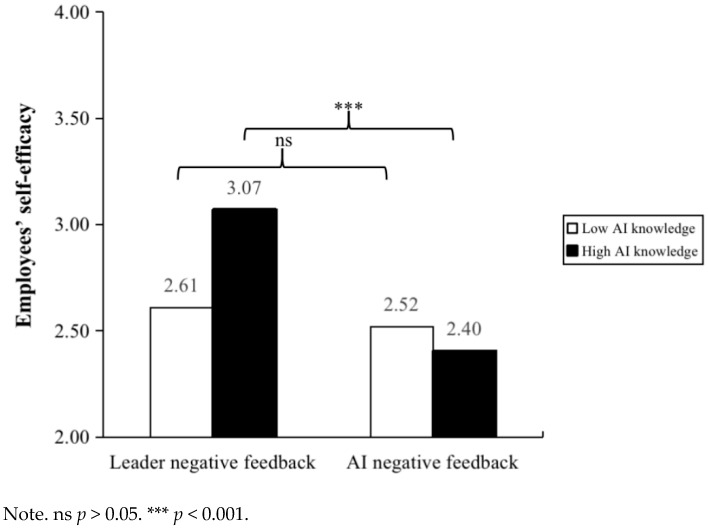
The moderating effect of employee AI knowledge between negative feedback source and employees’ self-efficacy in Study 3.

A 2 × 2 ANOVA with negative feedback source and employees’ AI knowledge as independent variables and employees’ feeling of shame as the dependent variable revealed a significant interaction effect of negative feedback source and employees’ AI knowledge, F _[1, 268]_ = 5.48, *p* < 0.05, η^2^ = 0.02. Furthermore, pairwise comparisons indicated that (see Table 2), given low employees’ AI knowledge, the leader negative feedback group’s feeling of shame (M = 3.15, SD = 0.11) was significantly higher than the AI negative feedback group’s (M = 2.50, SD = 0.12). Nevertheless, there is no significant difference between employees’ feeling of shame of the leader negative feedback group (M = 2.93, SD = 0.12) compared to employees’ feeling of shame of the AI negative feedback group (M = 2.82, SD = 0.11) given the high AI knowledge condition (see Figure 4). Thus, Hypothesis 5 was supported. A 2 × 2 ANOVA with negative feedback source and employees’ AI knowledge as independent variables and employees’ self-efficacy as the dependent variable revealed a significant interaction effect of negative feedback source and employees’ AI knowledge, F _[1, 268]_ = 5.82, *p* < 0.05, η^2^ = 0.02. Furthermore, pairwise comparisons indicated that (see Table 2), given high employees’ AI knowledge, the AI negative feedback group’s self-efficacy (M = 2.40, SD = 0.12) was significantly lower than the leader negative feedback group’s (M = 3.07, SD = 0.12). Nevertheless, there is no significant difference between employees’ self-efficacy of the leader negative feedback group (M = 2.61, SD = 0.12) compared to employees’ feeling of shame of the AI negative feedback group (M = 2.52, SD = 0.12) given the low AI knowledge condition (see Figure 5). Thus, Hypothesis 6 was supported.

We used SPSS 26.0 Hayes PROCESS (Model 7) to assess the conditional indirect effect of AI knowledge on the relationship between negative feedback source and employees’ withdrawal behavior via employees’ feeling of shame at low (−1 SD) and high (+1 SD) values of AI knowledge. The indirect effect was −0.16 (95% Boot CI = [−0.27, −0.06]) when employees’ AI knowledge was low, versus −0.03 (95% Boot CI = [−0.12, 0.05]) when employees’ AI knowledge was high. The index of moderated mediation was 0.13 (95% Boot CI = [0.02, 0.26]). Therefore, Hypothesis 7 was supported. We assessed the conditional indirect effect of AI knowledge on the relationship between negative feedback source and employees’ withdrawal behavior via employees’ self-efficacy at low (−1 SD) and high (+1 SD) values of AI knowledge. The indirect effect was 0.14 (95% Boot CI = [0.05, 0.25]) when employees’ AI knowledge was high, versus 0.02 (95% Boot CI = [−0.05, 0.10]) when employees’ AI knowledge was low. The index of moderated mediation was 0.12 (95% Boot CI = [0.02, 0.26]). Therefore, Hypothesis 8 was supported.

## 10. Discussion and Conclusions

Our research aimed to investigate the psychological and behavioral effects of negative feedback from leaders versus AI systems on employees. Specifically, we explored how negative feedback source influence employees’ feelings of shame, self-efficacy, and withdrawal behaviors, while considering the moderating role of employees’ AI knowledge.

We conducted three experiment studies to validate our theoretical model. Study 1 examined the relationship between negative feedback source and employees’ feelings of shame and self-efficacy. The results showed that leader negative feedback triggered stronger feelings of shame than AI negative feedback and AI negative feedback had a more significant negative impact on self-efficacy than leader negative feedback. However, Study 1 only tested part of our theoretical model and recruited only Chinese participants. Thus, we conducted Study 2 to test all the hypotheses.

Study 2, as well as revalidating the results of Study 1, also validated the indirect effect of negative feedback source on employee withdrawal behavior through employees’ feeling of shame and self-efficacy. Specifically, leader negative feedback induces stronger feelings of shame in employees compared to AI negative feedback, which subsequently leads to withdrawal behavior. In contrast, AI negative feedback has a greater negative effect on employees’ self-efficacy than leader negative feedback, prompting withdrawal behavior. Meanwhile, Study 2 investigated the moderating effects of employees’ AI knowledge. The findings revealed that for employees with low AI knowledge, leader negative feedback elicited stronger feelings of shame than AI negative feedback; however, this effect was not significant for those with high AI knowledge. Conversely, for employees with high AI knowledge, AI negative feedback had a more pronounced impact on reducing self-efficacy compared to leader negative feedback, while this effect was insignificant for employees with low AI knowledge.

In Study 2, AI knowledge was measured. In Study 3, we designed a between-subjects experiment with a 2 (leader negative feedback condition vs. AI negative feedback condition) × 2 (low AI knowledge condition vs. high AI knowledge condition) between-subjects design. Furthermore, we designed more immersive scenarios in Study 3 to enhance external validity. Study 3 also tested all the hypotheses.

### 10.1. Theoretical Implications

Our research has made significant contributions to the literature on AI and human feedback sources. First, our research delves into the distinct impacts of AI and leader negative feedback on employees, shedding light on the adverse effects of AI feedback. Previous research on AI feedback has predominantly focused on its beneficial effects on employees compared to human feedback. AI feedback is more personalized than human feedback, significantly boosting employee performance (Hoffmann & Thommes, 2020). Employees also view AI as offering more accurate evaluations, fostering a stronger sense of fairness (Qin et al., 2023). Additionally, AI’s real-time feedback improves employee commitment and satisfaction, while reducing turnover behaviors, more effectively than traditional leader feedback (Riaz & Ghanghas, 2024). Pei et al. (2024) found that, compared to human leader negative feedback, AI negative feedback can motivate employees’ promotion-focused cognition, impede their prevention-focused cognition, and thus enhance their job performance. In contrast to previous studies, our research reveals that the impact of AI feedback on employees does not always surpass that of human feedback. Specifically, when delivering negative feedback, our findings indicate that AI feedback can have a more detrimental effect on employee self-efficacy compared to feedback from human leaders, resulting in increased withdrawal behaviors. Our research explores the distinct impacts of AI and leader negative feedback, uncovering the “dark side” of AI feedback.

Second, we contribute to the literature on feedback sources by examining the varying effects of negative feedback from human leaders and AI. Feedback sources are critical factors influencing the psychological state and behaviors of feedback recipients (Herold et al., 1987). Current research on feedback sources mainly focuses on the effects of different sources such as organizations, colleagues, and leaders on feedback recipients (Greller & Herold, 1975; Hanser & Muchinsky, 1978; Ilgen et al., 1979). With the development of technology, AI has disrupted people’s perceptions of feedback sources, transitioning from a supportive tool to an active participant that plays a significant role in providing feedback within organizations (Lechermeier & Fassnacht, 2018; Tong et al., 2021). Moreover, AI symbolizes scientific accuracy and differs fundamentally from human leaders (Qin et al., 2023), thus necessitating the differentiation of the feedback effects between the two. However, there is a lack of systematic research on the differences in the effects of AI feedback and leader feedback on employees (Pei et al., 2024). Our research focuses on negative feedback and finds that AI negative feedback and leader negative feedback have different effects on employees through cognitive and emotional pathways. Our findings not only respond to scholars’ calls for clarifying the impact and mechanisms of AI as a feedback source on employees (Lechermeier & Fassnacht, 2018) but also highlight differences in different feedback sources (Herold et al., 1987), revealing distinctions in the effects of AI and human leaders’ negative feedback on employees.

Third, our research highlights the heterogeneity in individual employee perceptions of negative feedback sources, revealing the moderating effect of employees’ AI knowledge. This research clarifies who is more susceptible to the influence of AI negative feedback compared to leader negative feedback. We found that if employees have a greater understanding of AI, the effects of AI negative feedback are more likely to align with those of leader negative feedback, which can trigger feelings of shame. Additionally, with high AI knowledge, AI negative feedback (vs. leader negative feedback) tends to reduce their self-efficacy. This challenges the traditional view that AI knowledge is generally considered a favorable condition, wherein greater AI knowledge leads to more positive attitudes towards AI (Cao et al., 2021; Venkatesh et al., 2012). Previous research has mainly focused on employees’ individual perspectives, suggesting that possessing more knowledge about AI leads to its increased usage. However, when AI is applied to organizational processes, employees’ levels of AI knowledge play distinct roles. A deeper understanding of AI among employees leads them to perceive it as more objective and fairer (Langer & Landers, 2021), thereby amplifying the impact of AI negative feedback. Our research presents a comprehensive theoretical model that systematically explores the causal conditions of the mechanisms underlying the impact of negative feedback sources (AI vs. leaders) on employees.

### 10.2. Practical Implications

Our research reveals that negative feedback from AI can lead to adverse outcomes, underscoring the need for organizations to exercise caution when deploying AI feedback systems. This serves as a critical reminder that companies using AI-driven tools must prioritize an understanding of human psychology in their design. AI feedback mechanisms should not only provide accurate assessments but also deliver feedback in ways that enhance employees’ self-efficacy. Elements such as interface design, language choice, and feedback timing must be carefully considered to foster a positive work environment. In addition, organizations should establish clear guidelines on the appropriate use and limitations of AI in employee evaluations. By ensuring that technology complements, rather than replaces, human leadership, companies can achieve a balance between leveraging the efficiency of AI systems and maintaining the essential human element in employee management.

Moreover, to enhance the effectiveness of AI applications in organizations, managers must recognize the diverse roles that employees’ AI knowledge can play. Our research reveals that employees with greater AI knowledge experience more pronounced negative effects from AI negative feedback. In contrast, employees with lower AI knowledge are more adversely affected by leader-delivered negative feedback than by AI feedback. This highlights the need to consider employees’ subjective perceptions and individual differences when implementing AI feedback systems. For example, employees with advanced AI knowledge, such as those in technical roles, may respond more positively to negative feedback from leaders. On the other hand, employees with less AI knowledge and lower sensitivity to AI feedback, such as those in basic-function roles, may find AI-generated negative feedback more suitable. These insights can help organizations identify which employees benefit most from AI feedback systems, thereby refining their AI deployment strategies.

Lastly, employees must maintain an open mind when confronting negative feedback. Our findings indicate that AI negative feedback reduces employees’ self-efficacy more significantly than leader negative feedback, subsequently leading to work withdrawal behaviors. Conversely, leader negative feedback evokes stronger feelings of shame compared to AI negative feedback, which also contributes to work withdrawal behaviors. Thus, it is critical for employees to recognize the differing impacts of negative feedback depending on its source and to adopt proactive strategies to mitigate the adverse effects of negative cognitions and emotions on their work performance. For instance, when receiving AI negative feedback, employees can mitigate work withdrawal behavior stemming from decreased self-efficacy by concentrating on actionable suggestions for improvement provided in the feedback. In contrast, when receiving leader negative feedback, employees can channel feelings of shame into motivation to enhance their performance through effective positive emotion management strategies.

### 10.3. Limitations and Future Directions

There are several limitations in our research that warrant improvement in future research. First, our research identified the indirect effects of negative feedback sources on employee withdrawal behavior through feelings of shame and self-efficacy. Future research could expand on this by exploring additional negative emotions and cognitive responses that may influence these relationships. For example, leader negative feedback may induce more fear in employees and result in negative work behaviors compared to AI negative feedback, as leader feedback typically carries greater authority, potentially increasing stress and fear (Keltner et al., 2003). On the cognitive side, AI feedback may be perceived as less humanized and emotionally nuanced than leader feedback, which could lead employees to feel neglected or undervalued (Davenport & Ronanki, 2018).

Second, our research reveals that employees’ AI knowledge moderates the relationship between negative feedback sources and work withdrawal behaviors. Further research is needed to better understand the mechanisms through which AI negative feedback and leader negative feedback influence employee withdrawal behaviors. Potential moderators may include organizational environment, leadership characteristics, and individual differences such as AI aversion (Yam et al., 2022), which could amplify or reduce the impact of AI negative feedback on employee withdrawal behaviors. For instance, leaders’ high performance expectations, as noted by Judge and Bono (2001), may instill a sense of responsibility and trust in employees, promoting positive work behaviors and potentially mitigating the negative effects of feedback-related shame.

Third, concerning research methodology, we employed an experimental approach to validate all hypotheses. However, Study 2 and Study 3 faced limitations in measuring employee withdrawal behavior; specifically, we assessed withdrawal intentions rather than actual withdrawal behaviors. Furthermore, our research necessitated a comparative analysis of the impacts of AI negative feedback versus leader negative feedback, requiring both elements to be present in the research scenario. However, the simultaneous occurrence of these two elements in real-world settings is relatively rare, presenting significant challenges for field studies. Future research should explore work environments that fulfill these criteria. Conducting field studies using a multi-source, multi-wave design can enhance measurement accuracy and yield more robust findings.

Lastly, our research model was validated using both American and Chinese samples, demonstrating the robustness of the findings. However, future research could further explore how cultural factors moderate the effects of AI and leader feedback. Differences in leadership behaviors, feedback preferences, and technology acceptance across cultures (Euwema et al., 2007; Straub et al., 1997) may significantly influence the effectiveness of AI feedback. For instance, Eastern cultures, typically characterized by a high power distance (Hofstede, 1984), may exhibit greater reliance on traditional hierarchical feedback (Farh et al., 2007). In contrast, Western cultures, with their low power distance orientation, might be more receptive to the egalitarian, data-driven feedback approach provided by AI. Consequently, future studies should focus on the role of cultural factors in shaping the effectiveness of AI and leader feedback.

## Figures and Tables

**Table 1:** Hypothesis tests results in Study 2.

Variables	Shame		Self-Efficacy		Withdrawal Behavior
	Model 1	Model 2	Model 3	Model 4	Model 5
Predictors					
Negative feedback source	−0.42 **	−0.40 **	−0.83 ***	−0.80 ***	−0.34 *
AI knowledge		0.09		0.16 *	
Negative feedback source × AI knowledge		0.29 *		−0.45 **	
					
Shame					0.23 ***
Self-efficacy					−0.15 *
*R* ^2^	0.05	0.08	0.14	0.19	0.13
*F*	10.19 **	5.54 **	32.85 ***	15.58 ***	10.35 ***

Note. *n* = 207. Negative feedback source: 0 = negative feedback from leaders; 1 = negative feedback from AI. * *p* < 0.05. ** *p* < 0.01. *** *p* < 0.001 (two-tailed tests).

**Table 2:** Results of pairwise comparisons on employees’ feeling of shame and employees’ self-efficacy in Study 3.

			ΔMean	SE	*p*	95% CI	
						LL	UL
Shame	Low AI knowledge	Negative feedback from leaders vs. Negative feedback from AI	0.64	0.16	<0.001	0.32	0.96
	High AI knowledge	Negative feedback from leaders vs. Negative feedback from AI	0.10	0.16	>0.05	−0.22	0.43
Self-efficacy	Low AI knowledge	Negative feedback from leaders vs. Negative feedback from AI	0.09	0.17	>0.05	−0.24	0.42
	High AI knowledge	Negative feedback from leaders vs. Negative feedback from AI	0.67	0.17	<0.001	0.33	1.00

Note. *n* = 272. Negative feedback source: 0 = negative feedback from leaders; 1 = negative feedback from AI. AI knowledge: 0 = low AI knowledge; 1 = high AI knowledge.

## Data Availability

The data and syntaxes for the analyses of these three studies can be found on Open Science Framework: https://osf.io/9nkux/?view_only=c3f1b6214d454212b391c73242d89c8e (accessed on 18 April 2024).

## Note

1 We conducted a study (*n* = 330) to assess the relationship between AI knowledge and perceived AI performance effectiveness (Lu et al., 2019; Shi et al., 2021), and the results indicated a positive correlation between the two (r = 0.49, *p* < 0.001). Additionally, we also analyzed the correlation between AI knowledge and trust in AI (Merritt, 2011), confirming a positive relationship between the two (r = 0.55, *p* < 0.001).
